# Positive Effects of Non-Native Grasses on the Growth of a Native Annual in a Southern California Ecosystem

**DOI:** 10.1371/journal.pone.0112437

**Published:** 2014-11-07

**Authors:** Gregory J. Pec, Gary C. Carlton

**Affiliations:** Department of Biological Sciences, California State Polytechnic University, Pomona, California, United States of America; University of Saskatchewan, Canada

## Abstract

Fire disturbance is considered a major factor in the promotion of non-native plant species. Non-native grasses are adapted to fire and can alter environmental conditions and reduce resource availability in native coastal sage scrub and chaparral communities of southern California. In these communities persistence of non-native grasses following fire can inhibit establishment and growth of woody species. This may allow certain native herbaceous species to colonize and persist beneath gaps in the canopy. A field manipulative experiment with control, litter, and bare ground treatments was used to examine the impact of non-native grasses on growth and establishment of a native herbaceous species, *Cryptantha muricata*. *C. muricata* seedling survival, growth, and reproduction were greatest in the control treatment where non-native grasses were present. *C. muricata* plants growing in the presence of non-native grasses produced more than twice the number of flowers and more than twice the reproductive biomass of plants growing in the treatments where non-native grasses were removed. Total biomass and number of fruits were also greater in the plants growing in the presence of non-native grasses. Total biomass and reproductive biomass was also greater in late germinants than early germinants growing in the presence of non-native grasses. This study suggests a potential positive effect of non-native grasses on the performance of a particular native annual in a southern California ecosystem.

## Introduction

The decline of native plant species in disturbed areas has been linked to effects of competition for light and soil resources with non-natives [1], [2], [3]. Non-native grasses, in particular, are strong competitors that also tend to increase the frequency of wildfires [4], [5], [6]. Large portions of southern California shrublands, such as coastal sage scrub and chaparral, are being lost to non-native grass invasion [7]. Most coastal sage scrub and chaparral species are adapted to intense but infrequent fires [8]. In such conditions the ephemeral post-fire native community, consisting primarily of annual (60%) and perennial (20%) herbaceous species, is able to dominate for only one to three years before the shrub canopy closes [9]. With an increase in fire frequency, recruitment of fire-adapted native woody species may be hindered, slowing the formation of a closed woody canopy [9], [7]. Under these conditions non-native grasses and other herbaceous species persist longer after fire, and grasses may dominate patches in mature coastal sage scrub and chaparral communities [8]. However, the exact role of non-native grasses during recovery of these plant communities from fire remains unclear.

Positive interactions have received increased attention for their potential importance in structuring plant communities (see review by Booker et al. 2008). In general, a positive interaction occurs when a species is able to either improve the growth, survival or fitness of another (see review by Callaway 1995). A number of studies suggest that positive interactions can occur between native and non-native biota [10], [11], [12]. Non-native species can act as food sources or pollinators and can reduce predation pressure for native species (see examples in [11], [12]). Positive interactions can also occur between native and non-native plants, with the native species typically facilitating the growth and reproduction of the non-native species [13], [14], [15]. For example, Maron and Connors (1996) reported a positive effect of a nitrogen-fixing native shrub on the establishment of non-native species such as *Bromus diandrus* into coastal prairie plant communities [16]. Cavieres et al. (2005) provided evidence in a high alpine zone in central Chile for increased establishment of a non-native species on sites where the micro-environmental conditions had been modified by a native plant species [17]. Similarly, Griffith et al. (2010) showed that seedling establishment and reproductive potential of *Bromus tectorum* increased under native shrub microhabitats [18] and Zhang et al. (2011) reported a combined positive effect of two dominant native plant species on the performance of two associated non-native species [19]. Although these studies suggest that the positive effects of non-native plant species by natives may be fairly common, there is less evidence of a non-native plant species enhancing growth and reproduction of a native species [20], [21].

Positive interactions have also received attention as a potential vegetative restoration strategy for land management, particularly in disturbed areas [22], [23]. For example, Gómez-Aparicio et al. (2004) found that shrubs used as nurse plants enhanced the success of a reforestation project in a water-limited system. The shrubs provided a consistent positive effect on tree seedlings by increasing their survival and growth during four consecutive years [24]. Lugo (2004) reported that established non-native trees on degraded agricultural land in Puerto Rico provided positive effects in the restoration of native tree species. Non-native tree species rehabilitated soils and provided suitable microhabitat (e.g. light, air temperature) for native species to reestablish under these canopies [25]. In addition, identifying how less common native species respond to the effects of non-natives in disturbed systems may improve the success of restoration strategies for these systems [12].

The San Dimas Experimental Forest (SDEF) in the San Gabriel Mountains of southern California provides an ideal setting to study a possible role of non-native grasses in the persistence of native herbaceous species following fire. An intense wildfire burned across the entire experimental forest in 2002, and the recovering community still contains large areas of non-native grasses. In a previous study (Pec and Carlton, *unpublished data*), ordination and indicator species analysis showed that a native annual, *Cryptantha muricata,* was found only in sites that contained non-native grasses eight years following fire in recovering coastal sage scrub and chaparral communities within the SDEF (Appendix S1). That result prompted this follow-up study using a manipulative field experiment to test the effects of non-native grasses on *C. muricata.*

The goal of this study was to investigate possible positive effects of non-native grasses on the germination, survival, growth, and reproduction of *C. muricata*, a persisting native herbaceous species in a recovering shrub community. We addressed five questions.

1. Are specific environmental factors affected by the presence of non-native grasses?
2. Are germination and survival of *C. muricata* improved by the presence of non-native grasses?
3. Is growth of *C. muricata* enhanced by the presence of non-native grasses?
4. Is sexual reproduction of *C. muricata* increased by the presence of non-native grasses?
5. Are biomass allocation patterns of *C. muricata* affected by the presence of non-native grasses?

## Methods

### Study Species and Site

*Cryptantha muricata*, a member of the Boraginaceae family, is widely distributed throughout California. It is found on slopes of coastal sage scrub and chaparral communities and has characteristic inflorescences containing flowers that develop nutlets at maturity [26]. *C. muricata* is considered a fire-following species [9] that requires fire to germinate [27], but it has also been shown to germinate in high numbers without fire [28]. *C. muricata* is abundant in early post-fire years and is occasionally reported to persist in openings of mature chaparral and coastal sage communities [29], [9].

This study was conducted in the SDEF, located on the southern portion of the San Gabriel Mountains 45 km northeast of Los Angeles (latitude 34°19N and longitude 117°77W). Permits and approval for conducting the study on protected land were obtained from Michael Oxford, Forest Manager, United States Department of Agriculture, Forest Service, Pacific Southwest Research Station. The study area did not involve endangered or protected species.

The SDEF experiences a Mediterranean climate with cool, wet winters and warm, dry summers [30]. Following the most recent fire in September of 2002, upper to mid-level slopes in the SDEF have developed a mixture of hard-leaved sclerophyllous evergreen and associated soft-leaved drought-deciduous vegetation including *Adenostoma fasciculatum, Ceanothus* spp., *Eriodictyon* spp., *Eriogonum fasciculatum,* and *Salvia mellifera*. A few herbaceous species have persisted, with the most abundant being *Erodium cicutarium* and *Cryptantha muricata*. Several non-native grasses have also persisted at these elevations, including *Avena barbata, Bromus diandrus, Bromus madritensis* ssp. *rubens, Bromus tectorum, Ehrharta calycina* and *Festuca myuros*. No native grasses were observed on the study site. In 2011, cover of native vegetation and non-native grasses was 47% and 39%, respectively, with non-native grass cover dominated by *Bromus madritensis* ssp. *rubens*.

### Experimental Design

We used a fully randomized blocked design to test for the effects of non-native grasses on *C. muricata.* In January 2011, we randomly located and established twenty-five circular 28-m^2^ blocks on southwest-facing slopes of Bell Canyon (latitude 34°20N and longitude 117°77W), and on northeast-facing slopes of the West Fork of the San Dimas Canyon (latitude 34°12N and longitude 117°46W) at the SDEF (Appendix S1). Non-native grasses were abundant in all 25 blocks, and the proportional abundance of non-native grass species did not differ among blocks (Linear Mixed Model, *F* = 2.97, *P* = 0.084). Because of the similarity in non-native grass species composition in the micro-plots, differences in effects of individual non-native grass species on *C. muricata* were not tested. Three circular 1-m^2^ micro-plots were located randomly on each of the 25 blocks (Appendix S1). If a micro-plot was located in a portion of the block without 100% non-native grass cover, a new randomly chosen location was selected in the block. Each micro-plot was surrounded by a 1-m buffer zone.

The experiment consisted of three treatments, which were applied to micro-plots and the surrounding buffer zones. The **bare ground** treatment was created by cutting all grass stems 1 cm above the ground and removing cut grasses and existing litter. The **litter treatment** was created by cutting all grass stems 1 cm above the ground but placing the cut plants back in the micro-plot with the existing litter. The **control treatment** had no manipulation of non-native grasses. All micro-plots and surrounding buffer zones receiving the bare ground or litter treatment were maintained throughout *C. muricata's* growing season by periodic cutting of non-native grasses. Most of the emergence and active growth of non-native grasses occurred during late January and early February 2011 before *C. muricata* had germinated on any of the treatments. Emergence and growth of non-native grasses were negligible after March 2011, when daily temperatures rose and soil water began to decrease (G. Pec, *unpublished data and personal observation*).

Approximately 5000 seeds of the native forb, *Cryptantha muricata* (Hook. & Arn.) Nelson & J.F. Macbr, were obtained from Rancho Santa Ana Botanic Garden, Claremont, CA. These seeds were collected from a natural community growing at Yerba Buena Road, east of Circle × Ranch, Santa Monica Mountains, CA and from a wildflower bed in the Mesa plant community of the Rancho Santa Ana Botanic Garden, Claremont, CA (Michael Wall, *personal communication*). The average weight of 60 *C. muricata* seeds was determined by placing 15 sets of 60 seeds in the same Petri dish, weighing each set, and calculating the mean weight per set, which was 0.0182 g (±0.0004 SE). Then 75 sets of approximately 60 seeds, each set weighing 0.0182 g, were placed in small Petri dishes and parafilmed for transport. With 25 replicates, approximately 1,500 *C. muricata* seeds (25 replicates ×60 seeds/replicate) were applied to each treatment. Seeds were hand dispersed onto all 75 micro-plots during January 2011 when the soil was moist and there was little wind and no precipitation.

### Measurements

Environmental factors were measured at bi-weekly sampling periods during *C. muricata's* growing season from February to mid-May 2011 on each of the 75 micro-plots on the SDEF. During each sampling period, a LI-COR LI-1000 data logger with a thermistor probe was used to record soil surface temperature adjacent to each *C. muricata* germinant once during the morning and once during the afternoon. The same procedure was followed to record soil surface temperature at the center of each micro-plot containing no germinants. Mean values of the morning and afternoon readings were used to minimize bias due to diurnal changes in temperature. Soil water potential was estimated from five soil samples taken once during the morning and once during the afternoon just inside the edge of each micro-plot from the upper 10 cm of the soil column. Samples were placed in a plastic sample cup with lid (Decagon Devices, Pullman WA, USA), and sealed with parafilm for transport and storage. All samples were kept in refrigeration for one to two days and were then brought back to a constant temperature of 24°C at which time soil water potential measurements were taken using a WP-4 DewPoint PotentiaMeter (Decagon Devices, Inc., Pullman, WA, USA).

During each bi-weekly sampling period from February to mid-May 2011, germination rates and mortality data were recorded, and each new *C. muricata* germinant was tagged and assigned an identification number. During each sampling period, height, number of leaves, number of flowers, and number of inflorescences were also recorded for each tagged individual. In this species, number of inflorescences provides a fairly accurate estimate of the number of developing nutlets [26].

### Harvest Measurements

The last sampling period occurred during mid-May 2011 when all surviving *C. muricata* plants in each micro-plot had been flowering for about one month and fruits were developing with nutlets close to maturity on most plants. During the last sampling period all plants were harvested for biomass determination. Soil around each plant was watered prior to harvest. Individuals from the field were carefully removed with soil intact, placed into pots, transported, and sorted by treatment. Leaves of individuals from all treatments were collected and counted, and total leaf area for each plant was determined with an LI-3100 Leaf Area Meter (LI-COR, Lincoln, NE, USA). Senescing leaves were collected by plant before the final harvest and stored in refrigeration until the harvest, when they were combined with harvested leaves. Roots were separated from stems and washed with a 250-ml polyethylene wash bottle that allowed sufficient control of pressure and flow rate to retain fine roots. Reproductive structures were separated from stems, and all plant material was placed in separate paper bags. Individual parts were dried at 70°C for 48 h and weighed to determine biomass.

### Data Analysis

At least one individual *C. muricata* plant was found on 45 micro-plots at harvest, and 30 micro-plots did not contain any surviving individuals. On the three micro-plots that contained more than one individual *C. muricata* at harvest, a single individual was randomly chosen before data analysis to represent the treatment plot. To test for differences in germination rates between the three grass manipulation treatments, a linear mixed effects model was used with treatment and site (slope aspect) as fixed factors and block as a random factor. Linear mixed effects models with repeated measures were performed to test for effects of the grass manipulations on environmental factors experienced by *C. muricata* plants on the three treatments. If significant differences were found, Tukey-Kramer honestly significant difference (Tukey-Kramer HSD) tests were performed to determine which treatments were significantly different [31], [32].

Growth trends of *C. muricata* were tested for differences among the three treatments and across time. Orthogonal polynomials were used as weights to calculate linear and quadratic coefficients. Coefficients were then used as the raw data in individual linear mixed effects models with site as a fixed factor and block as a random factor [33], [34]. Log transformations were performed on height and number of leaves, and a square root transformation was performed on number of flowers and number of inflorescences. If significant differences were found, Tukey-Kramer HSD tests were performed to determine which treatments were significantly different [31], [32].

To test for effects of the three treatments on biomass and leaf area at final harvest, linear mixed effects models were used for total, shoot, stem, leaf, root and reproductive biomass and leaf area with treatment and site as fixed factors and block as a random factor in each of the models. Log transformations were performed on all variables except leaf area. Tukey-Kramer HSD tests were conducted to test for significant differences between specific treatments [31], [32]. Derived variables were calculated to identify allocation patterns in *C. muricata*. Stem mass ratio (SMR), leaf mass ratio (LMR), and root mass ratio (RMR) are the proportion of biomass allocated to stems, leaves, and roots, respectively [35]. Leaf area ratio (LAR) is the leaf area per unit total biomass [35]. Specific leaf mass (SLM) is the leaf mass per unit leaf area, an estimate of leaf thickness, and reproductive allocation (RA) is the reproductive biomass as a fraction of the total plant biomass [36], [35]. To test for treatment effects on allocation variables at final harvest, linear mixed effects models were used with treatment and site as a fixed factors and block as a random factor in each of the models. Log transformations were performed on SLM and RA. Tukey-Kramer HSD tests were conducted to test for significant differences between specific treatments [31], [32].

Differences in total and reproductive biomass at final harvest between early and late germinants in the control treatment were tested with individual linear mixed effects models. Allocation variables at final harvest between early germinants and late germinants in the control treatment were also tested with individual linear mixed effects models [32]. Site was used as a fixed factor and block was used as a random factor in each individual model. All data analyses were run using R 3.0.1, using the package *nlme* for all linear mixed effects models [37]. All model assumptions were checked by visual inspection of residual patterns [38].

## Results

**Figure 1:**
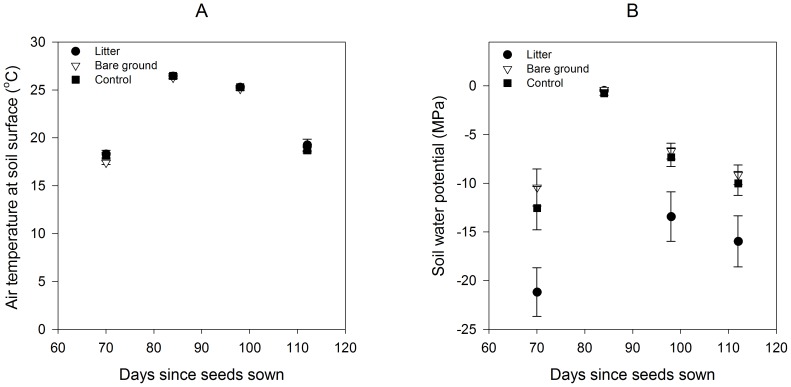
Environmental factors measured on each of three grass removal treatments in the San Dimas Experimental Forest. Panel (A) represents air temperature at the soil surface and panel (B) represents soil water potential on each of the three grass removal treatments (Control, *n* = 25, Litter, *n* = 25, Bare Ground, *n* = 25). Data are represented by means ±SE.

During the primary growing period, (70 to 112 days after seeds were sown) air temperature at the soil surface in the 75 micro-plots rose from day 70 to day 84 and then decreased (Fig. 1A). There was no significant difference among treatments or sites in overall air temperature from day 70 to 112 and no time-by-treatment interaction (Table 1, Fig. 1A). Soil water potential on the micro-plots also changed over time, becoming less negative from day 70 to day 84, following precipitation, but more negative from day 84 to day 112. There was no difference among sites, although there was a significant difference among treatments in overall soil water potential (Table 1). The litter treatment had a more negative soil water potential than both the bare ground and control treatments during the four growing periods, while the control treatment was not significantly different from the bare ground treatment (Fig. 1B). A significant time-by-treatment interaction reflected the uniform soil moisture across all treatments immediately following precipitation on day 84 (Table 1, Fig. 1B).

**Table 1:** Linear mixed effects models with repeated measures describing effects of three grass manipulation treatments on air temperature at the soil surface in °C and soil water potential in MPa over four growing periods (day 70 to day 112).

Effects	*F*	*P*
**Air temperature at the soil surface**		
Treatment _2, 252_	0.59	0.550
Time _3, 252_	108.67	**<0.0001**
Site _1, 252_	0.19	0.662
Treatment × Time _6, 252_	0.09	0.997
Treatment × Site _2, 252_	0.21	0.808
Time × Site _3, 252_	0.04	0.989
Treatment × Time × Site _6, 252_	0.59	0.737
**Soil water potential**		
Treatment _2, 252_	11.77	**<0.0001**
Time _3, 252_	13.30	**<0.0001**
Site _1, 252_	1.30	0.254
Treatment × Time _6, 252_	11.81	**<0.0001**
Treatment × Site _2, 252_	1.33	0.265
Time × Site _3, 252_	1.25	0.289
Treatment × Time × Site _6, 252_	1.283	0.265

**Figure 2:**
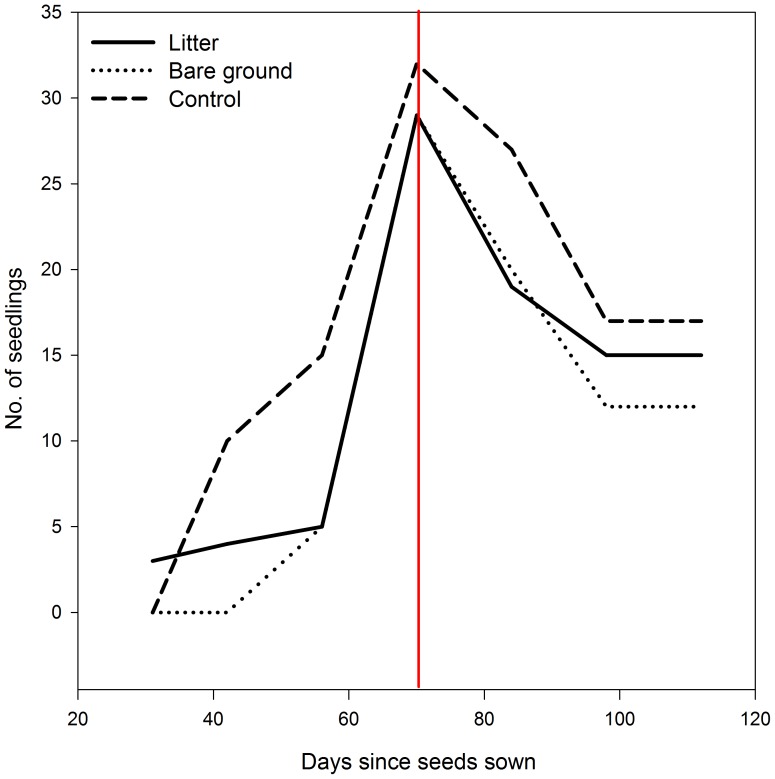
Number of live *Cryptantha muricata* on each of three grass removal treatments in the San Dimas Experimental Forest. Sample periods were from February to mid-May 2011. 1,500 seeds were hand dispersed per treatment on January 2011. 29 seeds germinated in the litter treatment, 32 in the control treatment, and 29 in the bare ground treatment. Red line represents where no further germination was observed on any plots after 10 weeks (day 70). Mortality of *C. muricata* was not observed until day 84 (right side of red line). All surviving individuals (Control, *n* = 17; Litter, *n* = 15; Bare Ground, *n* = 12) were harvested at 112 d.

A total of 90 individuals of *Cryptantha muricata* germinated on the three grass removal treatments (2% of the 4500 seeds sown). On all three treatments most of the germinants appeared 9 to 10 weeks after seeds were sown (Fig. 2). There was no difference in total germination rates (*F* = 0.04, *P* = 0.952) or site differences (*F* = 0.81, *P* = 0.390) among the three treatments. However, timing of germination did vary among the treatments. Earliest germination occurred on the litter treatment, with 3 seedlings appearing at the end of the first month (Fig. 2). Ten germinants were observed on the control treatment on day 42, 6 weeks after seeds were sown, and no individuals germinated on the bare ground treatment until day 56 (Fig. 2). By day 56, the control treatment had 15 germinants and the other two treatments had 5 each, but total germination was similar on all three treatments by day 70. No germination was observed on any plots after 10 weeks (day 70) (Fig. 2).

No mortality was observed until 84 days after seeds were sown (Fig. 2). Of the *C. muricata* that germinated, mortality was greatest on the bare ground treatment (58.6%) and least on the control treatment (46.9%)(Fig. 2). Greatest mortality was observed on day 84 on the litter and bare ground treatments and on day 98 on the control treatment. All individuals alive at day 98 survived until harvest at day 112 (Fig. 2).

**Figure 3:**
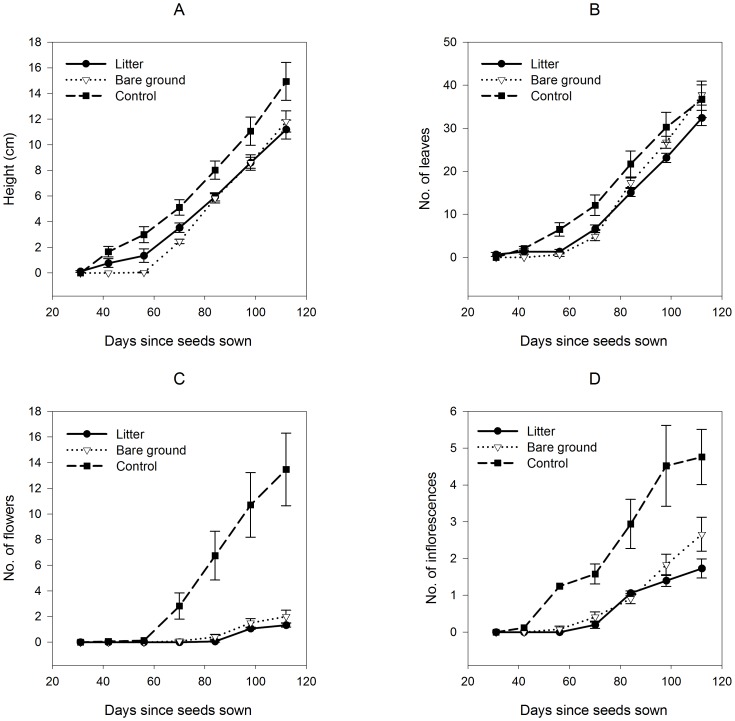
*Cryptantha muricata* growth measures on each of three grass removal treatments in the San Dimas Experimental Forest. Panel (A) represents height, (B) number of leaves, (C) number of flowers, and (D) number of inflorescences of *Cryptantha muricata* on each of three grass removal treatments across all sampling periods. Data are from plants that survived to harvest at day 112 and are represented by means ±SE.

Overall plant height differed significantly among treatments (Table 2 Total *F*), with plants in the control treatment taller throughout the experiment than those in the other treatments (Fig. 3A). From day 70 to day 112 mean height growth of *C. muricata* was linear on litter and bare ground treatments and quadratic with positive curvature on the control treatment (Fig. 3A, Table 2 Quadratic *F*). In other words, height growth rates from day 70 to day 112 were constant on the litter and bare ground treatments but increasing on the control treatment. The total number of leaves throughout the experiment did not differ significantly among the three treatments (Fig. 3B, Table 2 Total *F*). However, the slope of the curves for number of leaves differed between treatments, with the number of leaves increasing most rapidly in the bare ground treatment (Fig. 3B, Table 2 Linear *F*). The rate of leaf production in the control treatment declined toward the end of the experiment (Fig. 3B, Table 2 Quadratic *F*). The total number of flowers produced was significantly greater in the control treatment than the other treatments (Fig. 3C, Table 2 Total *F*). The rate of flower production increased much more rapidly in the control treatment than in the other two treatments (Fig. 3C, Table 2 Linear *F*). The negative curvature was statistically significant (Fig. 3C, Table 2 Quadratic *F*), but not nearly as significant biologically as the linear trend in number of flowers. As with flowers, the total number of inflorescences produced was much greater in the control treatment than in the bare ground or litter treatment (Fig. 3D, Table 2 Total *F*), with the litter treatment producing the fewest inflorescences. The rate of inflorescence production also increased most rapidly in the control treatment, and inflorescences were produced earlier in the control treatment than in the other treatments (Fig. 3D, Table 2 Linear *F*). The increase in number of inflorescences from day 70 to day 112 was quadratic with negative curvature for the control and litter treatments but fairly linear for the bare ground treatment (Fig. 3D, Table 2 Quadratic *F*).

**Table 2:** Summary of linear mixed effects models for total, linear, and quadratic contrasts describing growth of *Cryptantha muricata* over four growing periods.

	Total		Linear		Quadratic	
Source	*F*	*P*	*F*	*P*	*F*	*P*
† **Height**						
Grand Mean			184.47	**<0.0001**	71.68	**<0.0001**
Treatment	6.57	**0.002**	0.35	0.700	3.37	**0.039**
Site	0.62	0.431	0.00	0.930	0.53	0.468
† **Number of Leaves**						
Grand Mean			600.27	**<0.0001**	415.76	**<0.0001**
Treatment	1.87	0.157	3.94	**0.020**	3.92	**0.021**
Site	0.27	0.598	0.21	0.644	0.13	0.709
‡ **Number of Flowers**						
Grand Mean			47.11	**<0.0001**	48.21	**<0.0001**
Treatment	51.77	**<0.0001**	34.97	**<0.0001**	28.01	**<0.0001**
Site	0.05	0.813	0.12	0.729	0.02	0.872
‡ **Number of Inflorescences**						
Grand Mean			174.95	**<0.0001**	164.52	**<0.0001**
Treatment	23.02	**<0.001**	13.98	**<0.0001**	11.87	**<0.0001**
Site			0.15	0.690	0.21	0.645

*Notes*: Number of individuals per treatment - Control (*n* = 17), Litter (*n* = 15), Bare Ground (*n* = 12).

† Data were log-transformed.

‡ Data were square root transformed.

Biomass of *C. muricata* at final harvest differed greatly among the three field treatments. Plants in the control and bare ground treatments had significantly greater total biomass, stem biomass, root biomass, leaf biomass, and leaf area than plants in the litter treatment (Table 3, Appendix S1). Plants in the control treatment tended to have greater vegetative biomass than those in the bare ground treatment, but differences were not significant (Table 3, Appendix S1). Reproduction biomass was more than twice as great in the control treatment as the bare ground treatment and six times greater in the control treatment than the litter treatment (Table 3, Appendix S1).

**Table 3:** Comparison of biomass variables at final harvest (112 d) for *Cryptantha muricata* across three treatments.

	Treatment					
	Control (*n* = 17)		Litter (*n* = 15)		Bare Ground (*n* = 12)	
Growth Variable	Mean	±SE	Mean	±SE	Mean	±SE
†Total Biomass (mg) ***	^A^657.47	201.32	^B^138.66	20.86	^A^509.16	124.18
†Stem Biomass (mg) ***	^A^266.70	88.68	^B^40.13	6.41	^A^175.83	51.74
†Root Biomass (mg) **	^A^97.70	33.98	^B^21.86	4.69	^A^89.83	26.94
†Leaf Biomass (mg) **	^A^238.52	57.84	^B^67.93	17.53	^A^223.58	64.54
Leaf Area (cm^2^) **	^A^9.56	1.46	^B^3.63	0.53	^A^10.45	1.89
†Reproductive Biomass (mg) ****	^A^54.82	12.53	^C^9.00	0.85	^B^20.33	3.25

*Notes*: *n* =  number of harvested individuals per treatment. Significant differences were tested using Tukey-Kramer post-hoc tests.

**P<0.01,

***P<0.001,

****P<0.0001.

† Data were log-transformed.

Significant differences among the three treatments were also found in biomass allocation at final harvest (Table 4, Appendix S1). SMR was greatest for *C. muricata* grown in the control treatment and significantly differed from the litter treatment. RMR was not significantly different among treatments (Table 4, Appendix S1). LMR was greatest in the litter treatment and lowest in the control treatment (Table 4, Appendix S1). LAR and SLM did not differ significantly among treatments (Table 4, Appendix S1). Reproductive allocation was greater in the control treatment than either the litter or bare ground treatments (Table 4, Appendix S1).

**Table 4:** Comparison of allocation variables at final harvest (112 d) for *Cryptantha muricata* across three treatments.

	Treatment					
	Control (*n* = 17)		Litter (*n* = 15)		Bare ground (*n* = 12)	
Allocation Variable	Mean	±SE	Mean	±SE	Mean	±SE
(SMR) Stem Mass Ratio **	^A^0.370	0.016	^B^0.289	0.011	^AB^0.327	0.024
(RMR) Root Mass Ratio	0.131	0.008	0.147	0.008	0.176	0.038
(LMR) Leaf Mass Ratio **	^B^0.378	0.020	^A^0.490	0.013	^AB^0.440	0.027
(LAR) Leaf Area Ratio (cm^2^/g)	28.89	2.10	23.09	4.76	25.81	2.64
†(SLM) Specific Leaf Mass (g/cm^2^)	0.020	0.002	0.032	0.010	0.018	0.001
†(RA) Reproductive Allocation *	^A^0.119	0.018	^B^0.072	0.007	^B^0.056	0.009

*Notes*: *n* =  number of harvested individuals per treatment. Significant differences were tested using Tukey-Kramer post-hoc tests.

*P<0.05,

**P<0.01.

† Data were log-transformed.

**Figure 4:**
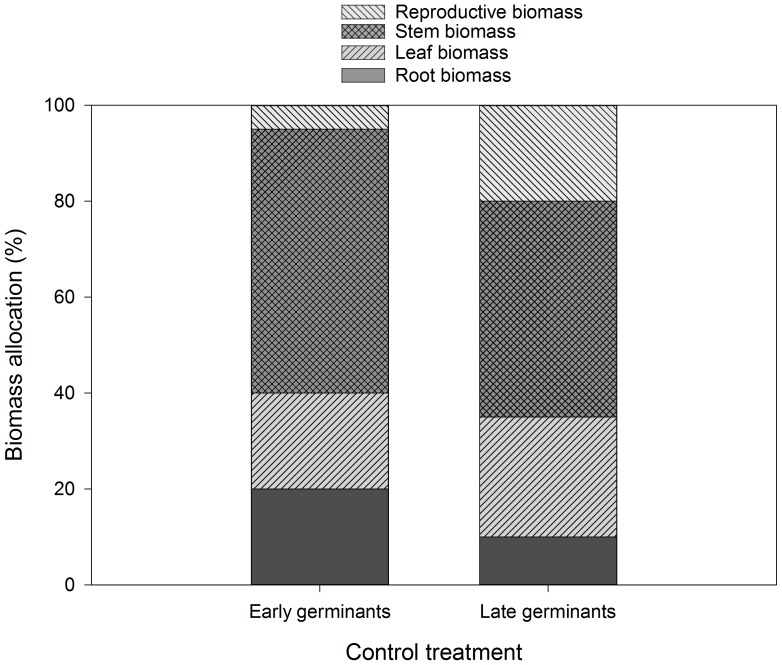
Biomass allocation for (*n* = 8) early and (*n* = 9) late germinants of *Cryptantha muricata* on the control grass removal treatment in the San Dimas Experimental Forest at final harvest (112 d).

Early germinants and late germinants in the control treatment differed significantly in total biomass, reproductive biomass, and biomass allocation at final harvest. Total biomass was much greater in late germinants (1050.00 mg±130.81 SE) than early germinants (308.33±13.33 SE)(*F* = 5.41, *P* = 0.03), and reproductive biomass was also greater in late germinants (67.50 mg±12.00 SE) than early germinants (31.00 mg±10.00 SE)(*F* = 8.61, *P* = 0.01). Early germinants allocated more to root biomass than late germinants (*F* = 5.34, *P* = 0.03), whereas late germinants allocated more to reproduction (*F* = 18.85, *P*<0.001) (Figure 4).

## Discussion

*Cryptantha muricata* plants surrounded in the control treatment by non-native grasses with 100% cover and height of 0.5 m±0.2 SE performed differently in many respects from those in plots where grasses were removed. Total germination was similar in all three treatments, but germination occurred earlier under the dense non-native grass canopy of the control treatment. The control treatment had three times as many germinants by the end of eight weeks as either of the other treatments. Mortality was lowest on the control treatment, but overall patterns of mortality were similar in all three treatments. Mortality began at a time when air temperatures near the soil surface rose across all plots. No mortality occurred during the last two weeks of the study, when temperatures had declined. During this time non-native grasses in the control treatment also began to senesce, providing a potential release for surviving late germinants of *C. muricata* to alter allocation patterns.

Soil water potential was not clearly related to mortality patterns. Mortality began when soils were dry before the precipitation recorded on day 84 and continued after day 84 when soil water was more available. During the last two weeks of the study when soils were again quite dry there was no mortality. Surprisingly, soil water potential was lowest in the litter treatment throughout the study period. The more negative soil water potential in the litter treatment may have been due to densely packed litter patches intercepting rainfall more effectively, thereby decreasing infiltration rates. Shading of the soil surface under non-native grasses may have led to higher water potential and increased survival in the control treatment [39]. Higher negative soil water potential in the bare ground treatment may have been due to a lack of vegetation, which increased water availability directly below the soil surface [40]. However, increases in soil surface temperature could have outweighed the potential benefit of increased soil water, leading to higher mortality rates for *C. muricata* in the bare ground treatment.

Plants in the control treatment differed from those in other treatments in growth and allocation patterns. This is not surprising because the proximity of neighbors can affect height, size, and reproductive allocation [41]. *C. muricata* plants in the control treatment were taller than those in the other treatments, but none of the *C. muricata* plants in any of the treatments exceeded the height of the tallest non-native grasses. Similar studies have shown that plants in crowded populations typically produce taller stems, often at the expense of diameter growth, than plants in uncrowded populations [42], [43]. Stem biomass and stem mass ratio were greatest for individuals of *C. muricata* growing in the presence of non-native grasses, but the greater total biomass of plants in the control treatment suggests that greater height growth did not require much sacrifice.

Crowded conditions may also lead to increased competition for belowground resources. In nutrient- and water-limited environments, plants often allocate more to root biomass and less to aboveground biomass [44]. In this study, *C. muricata* growing among grasses did not allocate more to roots than plants growing when grasses were removed, but because of their greater overall size, plants growing among grasses had more than four times the root biomass of plants growing in the litter treatment. Plants in the bare ground treatment attained roots nearly as large as those in the control treatment through slightly, although not significantly, greater allocation to roots. Root systems of many species have also been shown to redistribute water from lower to upper horizons through hydraulic lift [45], [46], [44]. Most grasses have been shown to concentrate about 60% of their roots in the first 10 cm of the soil profile. However, some grasses (e.g. *Bromus tectorum*) can reach rooting depths of up to 60 cm or more and also produce roots near the soil surface [47], [40]. The potential redistribution of water from lower to upper soil horizons by these grasses could increase water availability, decrease competition for water among shallow-root systems, and indirectly facilitate the availability of soil water and nutrients to neighboring species [48], [40]. Because soil water potential and root biomass of *C. muricata* were similar in the control and bare ground treatments, hydraulic lift by non-native grasses is probably not a significant cause of the enhanced growth and reproduction observed on the control treatment.

*Cryptantha muricata* reproduction was greatly enhanced in the presence of non-native grasses. *C. muricata* produced six times more flowers and also more inflorescences in the control treatment than in the treatments in which grasses were removed. Reproductive biomass was much greater in plants grown in the presence of non-native grasses, partly because of greater total biomass in these plants but also due to higher reproductive allocation in late germinants on the control treatment. These results differ from other studies in which survivorship, number of flowers, total seed mass, and total reproductive biomass per individual generally declined with increased plant density [49], [1].

*Cryptantha muricata* in the control treatment appeared to exhibit two different strategies for growth and fitness: (1) germinate early (in the first 8 weeks) and allocate more to root and stem biomass, or (2) germinate late (after 8 weeks), grow larger, and allocate less to root and stem biomass and more to reproductive output. Because of the greater number of early germinants in the control treatment (15 in the first 8 weeks compared to 5 in each of the other treatments) we thought that greater growth and reproduction in the control treatment may have been due largely to early germination. However, this was not the case. Surprisingly, plants that germinated late in the control treatment had three times greater reproductive biomass than those that germinated early due to the greater overall size and reproductive allocation of the late germinants. Germinating later may allow *C. muricata* to take better advantage of non-native grass senescence to exploit unused resources. Although both the early- and late-germinating *C. muricata* may have benefited from senescence of the grasses, the greater growth response of the late-germinating plants may have occurred because they were at an earlier stage in the life cycle when resources were released [50]. This difference in phenology may promote coexistence between the non-native grasses and *C. muricata*, as shown for other non-native and native species [51].

At local scales, native species, especially annual herbaceous species, may persist due to temporal or spatial heterogeneity in the physical environment [52], [53]. However, positive interactions may also play an important role in native annual establishment, growth, and survival. Few studies have found an interaction in which native plant species benefit from the presence of non-native species (but see [10], [12]). Our results demonstrate a positive effect of non-native grasses on the performance of a particular native annual, although care should be taken when interpreting removal experiments and their influence on species performance [54]. First, precipitation patterns, particularly in Mediterranean-type systems, are known to greatly affect herbaceous growth [55]. Variability in precipitation intra- or inter-annually can increase or reduce germination, survival and vigor of herbaceous annuals [50], [55], [56], while earlier winter precipitation can prompt early and more rapid non-native grass germination and establishment [57]. Native species might thus be under a phenological cue to germinate only when temperatures are cooler, precipitation is consistent, and photoperiod is shortened [50], [58]. Additionally, the litter and bare ground treatments could have created unfavorable conditions for *C. muricata*. Dense litter created by non-native grasses can reduce light availability at the soil surface, a factor that this study did not address. Slight increases in soil surface temperatures in the bare ground treatment may have altered seed germination and decreased seedling survival and growth of *C. muricata* [4], [59], [60]. Finally, multiple years of sampling will be required to detect the mechanisms behind the patterns observed from this single-year study, particularly if there is high year-to-year variability in abundance of non-native grass and native species and in availability of nutrients, particularly nitrogen [21], [56].

Our results do suggest that established non-native grasses could have potential for restoration of some degraded or disturbed wildland areas [61]. Established non-native grasses could be used to enhance the population size or preserve the seed bank of declining or less common native species, such as *C. muricata* [61]. For example, Elliott and Mackey (2008) found that a similar native plant species from the Boraginaceae family, *Cryptantha crinita,* although rare was able to persist in association with a number of non-native grasses in lowland and upland sites in northern California [62]. Additionally, in a previous study Pec and Carlton (*unpublished data*) found that eight years after fire non-native grasses were most abundant in the interface between chaparral and coastal sage scrub communities. Native species richness was also greatest in these transition zones. Additional research is thus needed to determine if positive effects on natives by non-natives may be more common than previously thought, particularly in transitional communities such as those we studied in the San Dimas Experimental Forest.

## Supporting Information

Appendix S1 **(1) Scatterplot of *Cryptantha muricata* abundance across three community types in the San Dimas Experiment Forest; (2) The San Dimas Experimental Forest located within the Angeles National Forest of Southern California; (3 and 4) Summary of linear mixed effects models testing biomass variables and allocation variables at final harvest (112 d) for *Cryptantha muricata*.**
(DOCX)
