# Supplementary material for: Positive Effects of Non-Native Grasses on the Growth of a Native Annual in a Southern California Ecosystem
Source: PLoS One. 2014 Nov 7;9(11):e112437. doi: 10.1371/journal.pone.0112437 (PMC4224496; doi:10.1371/journal.pone.0112437)
Supplement: Appendix S1 — (1) Scatterplot of Cryptantha muricata abundance across three community types in the San Dimas Experiment Forest; (2) The San Dimas Experimental Forest located within the Angeles National Forest of Southern California; (3 and 4) Summary of linear mixed effects models testing biomass variables and allocation variables at final harvest (112 d) for Cryptantha muricata. (DOCX) [file pone.0112437.s001.docx]

Appendix S1. (1) Scatterplot of *Cryptantha muricata* abundance across three community types in the San Dimas Experiment Forest; (2) The San Dimas Experimental Forest located within the Angeles National Forest of Southern California; (3 and 4) Summary of linear mixed effects models testing biomass variables and allocation variables at final harvest (112 d) for *Cryptantha muricata*.


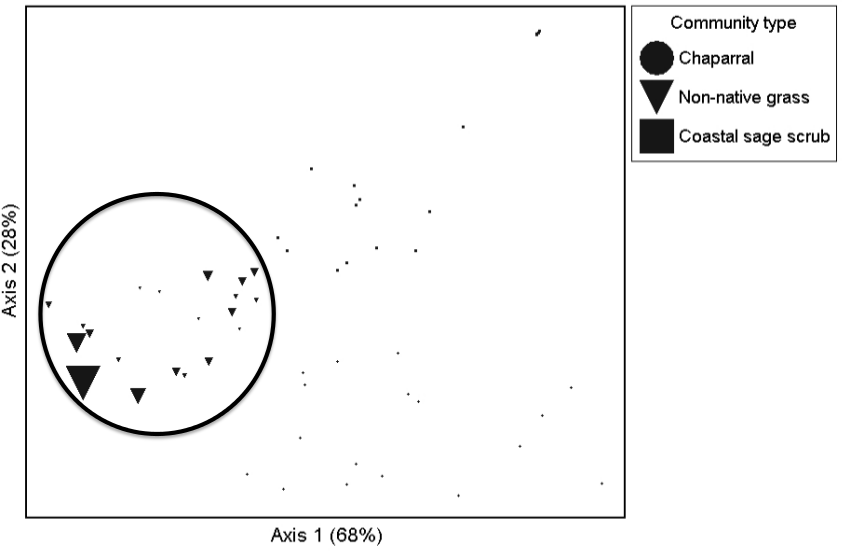


1. Scatterplot of *Cryptantha muricata* abundance across three community types in the San Dimas Experimental Forest. Each point represents a site with symbols to indicate community type. Axis 1 and axis 2 explain 68% and 28% of the variance, respectively. Symbol size is proportional to *C. muricata’s* abundance across all community types. Circle encloses the presence of *C. muricata,* which is found only in the non-native grass community identified by non-metric multidimensional scaling ordination.


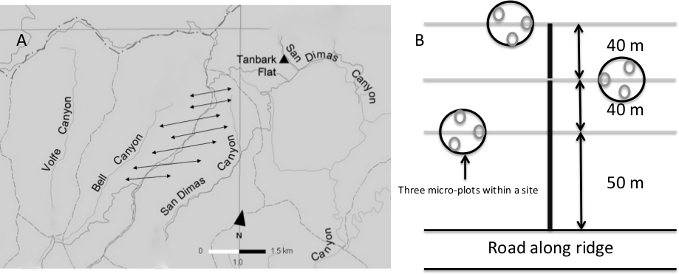


2. (A) The San Dimas Experimental Forest located within the Angeles National Forest of Southern California. Each of seven lines represents one transect on southwest-facing slopes toward Bell Canyon and one transect on northeast-facing slopes toward the West Fork of San Dimas Canyon. (B) Representative random location of sites along a transect line. Along each transect, micro-plots are located at 40-m intervals at random offsets from the transect line. To minimize the effects of human disturbance and non-native populations along the roadside, a starting distance of 50 m was used.

3. Summary of linear mixed effects models testing biomass variables at final

harvest (112 d) for *Cryptantha muricata*.

|  | *F* | *P* |
| --- | --- | --- |
| Total Biomass |  |  |
| Treatment _2, 22_ | 9.046 | **<0.001** |
| Site _1, 22_ | 0.072 | 0.790 |
| Treatment x Site _2, 22_ | 0.092 | 0.912 |
|  |  |  |
| Stem Biomass |  |  |
| Treatment _2, 22_ | 10.841 | **<0.001** |
| Site _1, 22_ | 0.043 | 0.837 |
| Treatment x Site _2, 22_ | 0.114 | 0.892 |
|  |  |  |
| Root Biomass |  |  |
| Treatment _2, 22_ | 5.824 | **0.009** |
| Site _1, 22_ | 0.185 | 0.670 |
| Treatment x Site _2, 22_ | 0.187 | 0.830 |
|  |  |  |
| Leaf Biomass |  |  |
| Treatment _2, 22_ | 6.049 | **0.008** |
| Site _1, 22_ | 0.169 | 0.684 |
| Treatment x Site _2, 22_ | 0.105 | 0.900 |
|  |  |  |
| Leaf Area |  |  |
| Treatment _2, 22_ | 8.185 | **0.002** |
| Site _1, 22_ | 0.001 | 0.966 |
| Treatment x Site _2, 22_ | 0.156 | 0.855 |
|  |  |  |
| Reproductive Biomass |  |  |
| Treatment _2, 22_ | 16.028 | **<0.0001** |
| Site _1, 22_ | 0.219 | 0.644 |
| Treatment x Site _2, 22_ | 0.390 | 0.681 |

*Note*: Data were log-transformed for all growth variables except Leaf Area.

4. Summary of linear mixed effects models testing allocation variables at final

harvest (112 d) for *Cryptantha muricata*.

|  | *F* | *P* |
| --- | --- | --- |
| (SMR) Stem Mass Ratio |  |  |
| Treatment _2, 22_ | 6.133 | **0.007** |
| Site _1, 22_ | 3.626 | 0.070 |
| Treatment x Site _2, 22_ | 1.732 | 0.200 |
|  |  |  |
| (RMR) Root Mass Ratio |  |  |
| Treatment _2, 22_ | 1.240 | 0.308 |
| Site _1, 22_ | 0.950 | 0.340 |
| Treatment x Site _2, 22_ | 1.308 | 0.290 |
|  |  |  |
| (LMR) Leaf Mass Ratio |  |  |
| Treatment _2, 22_ | 7.421 | **0.003** |
| Site _1, 22_ | 1.125 | 0.300 |
| Treatment x Site _2, 22_ | 0.023 | 0.976 |
|  |  |  |
| (LAR) Leaf Area Ratio |  |  |
| Treatment _2, 22_ | 0.998 | 0.386 |
| Site _1, 22_ | 0.004 | 0.947 |
| Treatment x Site _2, 22_ | 0.095 | 0.908 |
|  |  |  |
| (SLM) Specific Lead Mass |  |  |
| Treatment _2, 22_ | 1.362 | 0.276 |
| Site _1, 22_ | 0.171 | 0.682 |
| Treatment x Site _2, 22_ | 0.027 | 0.972 |
|  |  |  |
| (RA) Reproductive Allocation |  |  |
| Treatment _2, 22_ | 3.408 | **0.040** |
| Site _1, 22_ | 1.213 | 0.281 |
| Treatment x Site _2, 22_ | 0.103 | 0.902 |

*Note*: Data were log-transformed for Specific Leaf Mass and Reproductive Allocation.
